# The Mitochondrial Genome of the Glomeromycete *Rhizophagus* sp. DAOM 213198 Reveals an Unusual Organization Consisting of Two Circular Chromosomes

**DOI:** 10.1093/gbe/evu268

**Published:** 2014-12-19

**Authors:** Maryam Nadimi, Franck O.P. Stefani, Mohamed Hijri

**Affiliations:** Département de Sciences Biologiques, Université de Montréal, Institut de Recherche en Biologie Végétale (IRBV), Quebec, Canada

**Keywords:** mitochondrial genome, genome sequencing, basal fungal lineages, fungi, plasmid-like DNA polymerase genes (*dpo*), arbuscular mycorrhizal fungi, *Glomeromycota*, *Rhizophagus*

## Abstract

Mitochondrial (mt) genomes are intensively studied in *Ascomycota* and *Basidiomycota*, but they are poorly documented in basal fungal lineages. In this study, we sequenced the complete mtDNA of *Rhizophagus* sp. DAOM 213198, a close relative to *Rhizophagus irregularis*, a widespread, ecologically and economical relevant species belonging to *Glomeromycota*. Unlike all other known taxonomically close relatives harboring a full-length circular chromosome, mtDNA of *Rhizophagus* sp. reveals an unusual organization with two circular chromosomes of 61,964 and 29,078 bp. The large chromosome contained nine protein-coding genes (*atp9*, *nad5*, *cob*, *nad4*, *nad1*, *nad4L*, *cox1*, *cox2*, and *atp8*), small subunit rRNA gene (*rns*), and harbored 20 tRNA-coding genes and 10 *orf*s, while the small chromosome contained five protein-coding genes (*atp6*, *nad2*, *nad3*, *nad6*, and *cox3*), large subunit rRNA gene (*rnl*) in addition to 5 tRNA-coding genes, and 8 plasmid-related DNA polymerases (*dpo*). Although structural variation of plant mt genomes is well documented, this study is the first report of the presence of two circular mt genomes in arbuscular mycorrhizal fungi. Interestingly, the presence of *dpo* at the breakage point in intergenes *cox1-cox2* and *rnl-atp6* for large and small mtDNAs, respectively, could be responsible for the conversion of *Rhizophagus* sp*.* mtDNA into two chromosomes. Using quantitative real-time polymerase chain reaction, we found that both mtDNAs have an equal abundance. This study reports a novel mtDNA organization in *Glomeromycota* and highlights the importance of studying early divergent fungal lineages to describe novel evolutionary pathways in the fungal kingdom.

## Introduction

Mitochondria are membrane-bound organelles that are involved in several cell processes such as adenosine triphosphate (ATP) production via oxidative phosphorylation, respiration, RNA maturation, and protein synthesis. Mitochondria are also involved in cell division, growth, and death. They harbor their own genetic material that has evolved from an ancestral prokaryote genome. The endosymbiotic theory (Margulis 1971) suggests that the origin of nuclear genome of eukaryotic cells evolved in parallel to the origin of mitochondrial (mt) genome (Gray et al. 1999; Lang et al. 1999). Structure, size, and even function of mt genomes are variable among eukaryotes (Fukuhara et al. 1993; Drissi et al. 1994; Nosek and Tomaska 2003; Alverson et al. 2011; Nadimi et al. 2012; Beaudet, Nadimi, et al. 2013). Previous studies have shown that mitochondria exhibit a large diversity of genome architectures. For example, linear, circular, and fragmented mtDNAs have been described in *Cucumis*, *Phythium*, Ichthyosporean protists, *Globodera pallida*, *Pediculus humanus capitis*, *Candida labiduridarum*, *Candida frijolesensi*s, and *Brachionus plicatilis* (Martin 1995; Armstrong et al. 2000; Burger et al. 2003; Suga et al. 2008; Alverson et al. 2011; Valach et al. 2011; Shao et al. 2012). Genome reshuffling and evolution of mtDNA structures have been observed in phylogenetically distant eukaryotic lineages as well as in closely related species (Fukuhara et al. 1993; Wilson and Williamson 1997). Conversion of circular genomes to monomeric linear genomes has been shown to occur by an insertion of linear plasmids with inverted terminal repeats (Schnare et al. 1986; Heinonen et al. 1987), resulting in the extension of mt genome size. Another feature of mtDNA is the size variability among eukaryotic lineages, spanning from ∼6 kb in *Plasmodium falciparum* (*Apicomplexa*) to 11.3 Mb in the angiosperm genus *Silene* (Conway et al. 2000; Sloan et al. 2012). Mitochondria without any genes and organisms lacking mitochondria have been reported (reviewed in Keeling and Slamovits 2004). The gene content of mtDNA also varies broadly from 5 genes in *Plasmodium* (Conway et al. 2000) to 100 genes in jakobid flagellates (Burger et al. 2013), while 40–50 genes are commonly observed in mtDNA of eukaryotes. Gene content, size of introns, intergenic regions, and mobile elements such as open reading frames (*orfs*), plasmid-related DNA polymerase sequences (*dpo*), and short inverted repeats (SIRs) are the major causes of polymorphism in mt genomes of eukaryotes.

Arbuscular mycorrhizal fungi (AMF) are members of the phylum *Glomeromycota* (Schüssler et al. 2001) and they represent an early-diverging fungal lineage dating back to the Early Devonian (Remy et al. 1994; Redecker et al. 2000). AMF are plant root-inhabiting fungi, where they form mutualistic symbiotic associations with ∼80% of vascular plants (Smith and Read 2008). They promote plant growth by enhancing mineral uptake, in particular phosphorus, and protect plants against pathogens by controlling the growth of some soil fungal pathogens or by inducing plant defense responses (Ismail et al. 2011, 2013; Ismail and Hijri 2012). Recently, nuclear and mitochondrial genomics of AMF have been intensively studied (Tisserant et al. 2012, 2013; Halary et al. 2013). The first published mt genome of AMF was *Rhizophagus irregularis* isolate 494, (previously named *Glomus intraradices* and then *Glomus irregulare*) followed by the publication of the mt genomes of 11 taxa belonging to the genera *Rhizophagus*, *Glomus*, and *Gigaspora* (Lee and Young 2009; Formey et al. 2012; Nadimi et al. 2012; Pelin et al. 2012; Beaudet, Nadimi, et al. 2013; Beaudet, Terrat, et al. 2013; de la Providencia et al. 2013). AMF identification using the traditional ribosomal DNA markers of the nuclear genomes is uncertain due to high levels of intraspecific variations (Stockinger et al. 2009; Kruger et al. 2012; Schoch and Seifert 2012). Therefore, the publication of mt genomes provides useful data to identify AMF strains. For instance, sequences from intergenic and intronic regions are very divergent, which allows discrimination of closely related isolates (Formey et al. 2012; Beaudet, Terrat, et al. 2013; de la Providencia et al. 2013).

Mitochondrial genome sequencing provides insights into the mtDNA evolution within *Glomeromycota*. Indeed, mtDNA structure in *Glomeromycota* has been shown to undergo different evolutionary mechanisms such as fragmented genes (Nadimi et al. 2012), lateral gene transfer, insertion/excision of mobile elements (Beaudet, Nadimi, et al. 2013; Beaudet, Terrat, et al. 2013), and transmission of SIRs (Formey et al. 2012; Beaudet, Terrat, et al. 2013). AMF mt genomes have been invaded by different types of selfish mobile genetic elements (MGEs) or mobilomes, such as homing endonuclease, plasmid-related DNA polymerase (*dpo*), and SIRs (Formey et al. 2012; Beaudet, Terrat, et al. 2013). However, their movement and recombination mechanisms are not clearly understood. MGEs are typically known as DNA fragments encoding enzymes and other proteins that mediate the movement of the related chromosomal segment within genomes (intracellular mobility) or between different individuals (intercellular mobility). SIRs are thought to cause a double-strand breakage (DSB) in close vicinity resulting in initiation of their replication (Rattray et al. 2001). Pairing of two similar SIRs can lead to intrachromosomal homologous recombination resulting in genome rearrangements (Smith 1988; Romero and Palacios 1997).

The plasmid-related DNA polymerase (*dpo*) genes typically found in mitochondria are believed to be of a bacterial origin (Griffiths 1995; Cermakian et al. 1997). Mitochondrial plasmids are small extragenomic mtDNA molecules that can be transmitted vertically and horizontally, increasing the probability of gene transfer between genetically distinct mitochondrial genomes (Yang and Griths 1993). *dpo* genes have been found in mtDNA of many fungi such as *Glomeromycota*. Using comparative mt genomics, Beaudet, Terrat, et al. (2013) reported evidence of *dpo*-mediated interhaplotype recombination leading to mt genome rearrangement in *Rhizophagus* sp. DAOM 240422. This study revealed the presence of highly similar plasmids in distantly related fungal lineages, supporting horizontal gene transmission of these elements. However, it is not yet clear how *dpo* sequences are integrated into mtDNA and how they move within mtDNAs.

In this study, we report a novel mtDNA organization in the *Rhizophagus* sp. isolate DAOM 213198, a close relative of the model AMF *R. irregularis*. Using bioinformatic analyses and experimental evidence, we characterize the unusual multichromosomal architecture of mtDNA in *Glomeormycota*. This peculiar mtDNA architecture provides the opportunity to assess the evolutionary process on mtDNA in *Glomeromycota* and the mechanism by which the fragmentation of mtDNA occurs. The mt genome of *Rhizophagus* sp. DAOM 213198 provides insights into the role of *dpo* and SIRs in mtDNA structure and evolution.

## Materials and Methods

### Fungal Material and DNA Extraction

*Rhizophagus* sp. DAOM 213198 (synonym MUCL 43203) was obtained from the National Mycological Herbarium (DAOM), Ottawa, ON, Canada. This isolate was isolated from the perennial grass *Agrostis gigantean* collected from Quetico Provincial Park, ON, Canada, in 1989. Spore morphology showed some similarities with *R. irregularis* (supplementary fig. S1, Supplementary Material online). The isolate DAOM 213198 is closely related to *R. irregularis* (molecular virtual taxon VTX00114, accession number: AM849267) because the pairwise alignment of their 18S rDNA sequences has 99% of similarity (13 different nucleotide substitutions out of 1,716 bp).

The isolate DAOM 213198 was cultivated *in vitro* on a minimal (M) medium with carrot roots transformed with *Agrobacterium rhizogenes*. The growing medium containing mycelium and spores was dissolved in extraction buffer (0.82 mM sodium citrate and 0.18 mM citric acid). DNA was extracted from spores and hyphae using the DNeasy Plant Mini Kit (Qiagen, Toronto, ON) according to the manufacturer’s recommendations, except that spores and hyphae were crushed using a micropestle in a 1.5-ml tube containing 400 µl of AP1 buffer.

### Sequencing and Bioinformatic Analyses

Total DNA from *Rhizophagus* sp*.* DAOM 213198 was sequenced using Roche 454 GS FLX Titanium. The full sequence run and subsequent Sanger sequencing were performed at the Genome Quebec Innovation Center (McGill University, Montreal, QC). Reads were *de novo* assembled with Newbler software (Roche, Version 2.9) at the Genome Quebec Innovation Center. Gene annotation was performed automatically with MFannot (http://megasun.bch.umontreal.ca/cgi-bin/mfannot/mfannotInterface.pl, last accessed December 16, 2014), followed by manual inspection and editing as described in Nadimi et al. (2012). The mtDNA of *R. irregularis* DAOM 234179 (KC164354) was used as a reference for comparative analyses. This isolate has been chosen as a reference because of its common gene synteny among Glomeromycetes (Beaudet, Terrat, et al. 2013). Polymerase Chain reaction (PCR) and Sanger sequencing were performed to fill the gaps and join mtDNA contigs. The annotated sequences of the mt circular chromosomes of *Rhizophagus* sp*.* DAOM 213198 were deposited in GenBank under accession numbers KF591215 and KF591216, respectively. mtDNA circular maps were built using OGDRAW Version 1.2 software (Lohse et al. 2013). Sequence and comparative analyses were performed using the National Center for Biotechnology Information (NCBI) genomic database and multiple sequence alignment. Sequences were compared at the amino acid level due to high mutation and substitution rates in intergenic regions and *dpo*-like sequences. A search with TBLASTX was used to find the translated nucleotide database in NCBI using a minimum *E*-value cutoff of 1 × 10^−^^10^ and 50% minimum identity.

### Polymerase Chain Reactions

Conventional and long-range PCRs were used to sequence gaps between contigs, to validate gene synteny, to demonstrate the circularity of the small mt chromosome (primers are listed in table 1), and to correct potential pyrosequencing errors in homopolymer stretches. Conventional PCRs were performed in a final volume of 50 µl containing 1X PCR buffer, 1.5 mM MgCl_2_, 0.2 mM of each deoxynucleotide triphosphate, 0.5 µM of each primer, and 1 unit of Platinum *Taq* DNA Polymerase (Life Technologies, Burlington, ON). Cycling parameters were as follows: 94 °C for 90 s, followed by 38 cycles of 94 °C for 1 min, annealing temperature (table 1) for 30 s, 72 °C for 90 s, and a final elongation at 72 °C for 5 min. Long-range PCR reactions were also performed to attempt to assemble the last two mt contigs. Long-range PCR reactions were done in a final volume of 50 µl using TaKaRa LA PCR Amplification Kit (Clontech, Mountain View, CA) according to the manufacturer’s instructions or using the PCR protocol for LongAmp Hot Start Taq DNA Polymerase (New England Biolabs). PCRs were run in an Eppendorf Mastercycler ProS (Eppendorf, Mississauga, ON). PCR products were separated by electrophoresis in a 1% or 0.8% (w/v) agarose gel, stained with GelRed Nucleic Acid Gel Stain (Biotium Inc., Hayward, CA) and visualized with a Gel Doc System (BioRad, Mississauga, ON).

**Table 1:** Primers and Probes Designed for the Real-Time qPCR Assays and Long-Range PCR Primers Used to Validate the Circularity of the Two mtDNAs in *Rhizophagus* sp*.* DAOM 213198

Region and Primer Name	Primer Direction	Sequence (5′–3′)	Size (bp)	*T*_m_ (°C)
*cox1*	F	CTTCGCAGGTTGGAATATG	5,690	60
*cox2*	R	CATCCAGCCAACACCTAGTAG		59
*Rnl*	F	ATAGGACCTGGGTGTAATAGC	3,309	58
*atp6*	R	GCCTATATTAGTCAGCCCA		56
*8.1*	F	GGACAAATACGCTACTCTTATAGATTCAGAAC	9,911	65.1
*6.2*	R	GCTTCAAACATTCTGATGAGGCTAAAGA		65.6
*7.1*	F	GGAGAGTAGGTTTTAAACAGTGTCTAATTTCTG	9,100	65.6
*8.2*	R	GTTCTGAATCTATAAGAGTAGCGTATTTGTCC		65.1
*6.1*	F	TCTTTAGCCTCATCAGAATGTTTGAAGC	1,0160	65.6
*7.2*	R	CAGAAATTAGACACTGTTTAAAACCTACTCTCC		65.6
*nad4*	F	TCTTCGTGCTTCTGACTAC	125	55.5
	R	GTCTCTATCACAAGTAGCGA		54.4
	Probe	TCCGGTAATATTTCCGCTGTCAA		65
*cox3*	F	ATGAAGTTTCAACCTCATCCTTATC	126	62.2
	R	TCCTCCATGACCATATCCATG		64.5
	Probe	AGTAGAACCTTCACCATGGCCTCTAGCA		71.7
*rnl* intron 4	F	GACTTTACGTGGTTCTAGTTGTTAG	150	60.6
	R	CTACCCTAGTAAGTAAGGGTTTGG		60.9
	Probe	TGAAACAATTGGGTTCAAATCAAGGGTTGT		67.1

Note.—Primer direction (F, forward and R, reverse), sequence, PCR product size in base pair (bp), and melting temperature in degree Celsius (*T*_m_) are indicated.

### Quantitative Real-Time PCR

The abundance of each mt genome in DAOM 213198 was assessed by quantitative real-time PCR (qPCR). Four specific TaqMan assays (table 1) targeting intron 4 of *rnl* and *cox3* genes for the small mt chromosome and intron 7 of *cox1* and *nad4* genes for the large mt chromosome were designed to quantify the abundance of each chromosome. TaqMan probes labeled with 5′ FAM fluorophore and 3′ BHQ-1 quencher (Alpha DNA, Montreal, QC) and primers were designed using the plugin Primer3 (Untergrasser et al. 2012) implemented in Geneious Version 7.1.4 (BioMatters, Auckland, NZ). The cycling parameters for qPCR were 95 °C for 3 min, followed by 40 cycles of 95 °C for 15 s and the final elongation at 60 °C for 1 min. qPCRs were performed in a final volume of 20 μl containing 10 μl of iTaq Universal Probes Supermix (Bio-Rad Laboratories, Mississauga, ON), 0.65 μM of each primer, 0.08 μM of TaqMan probe, and 2.7 μl of DNA template. Four and three 10-fold serial dilutions were used as DNA templates for DAOM 213198 and 197198, respectively. Isolate DAOM 197198 was used for comparison purposes. Absolute quantification was performed using six 10-fold serial dilutions of circular plasmids. DNA was quantified using the Qubit dsDNA BR and HS Assay Kits (Life Technologies) and the Qubit 2.0 Fluorometer (Life Technologies) according to the manufacturer’s instructions. Each sample was amplified in triplicate in a ViiA 7 Real-Time PCR System (Life Technologies). PCR efficiency was calculated by converting the slope produced by the linear regression of the curves to percentage efficiency using the formula: Efficiency = −1 + 10 (−1/slope).

## Results and Discussion

### Description of *Rhizophagus* sp. mtDNA

**Fig. 1.—:**
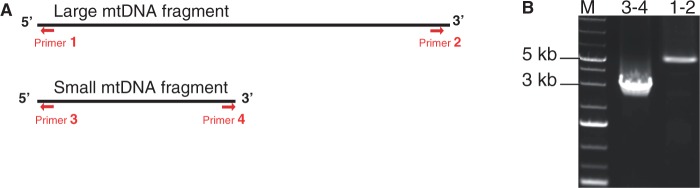
(*A*) Schematic representation of the two mtDNA contigs obtained after 454 reads assembly of *Rhizophagus* sp. DAOM 213198. Red arrows represent the position and orientation of primers shown in table 1. (*B*) Agarose gel electrophoresis showing long-range PCR amplification patterns using primers designed in the termini of each contig. The sizes of the amplicons were 5,690 and 3,309 for “1–2” and “3–4” primer combinations, respectively. M, molecular marker.

**Fig. 2.—:**
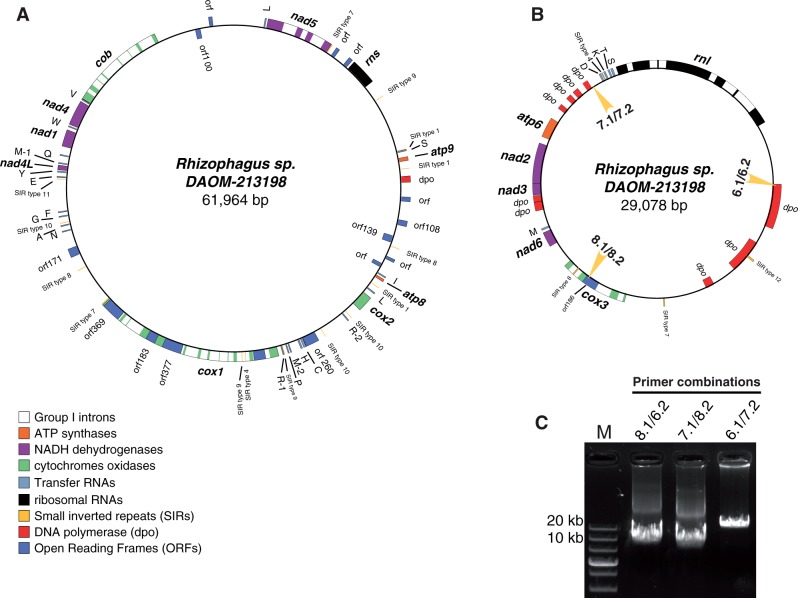
mtDNA circular maps of *Rhizophagus* sp. DAOM 213198. The mt genome consists of two circular-mapping chromosomes (*A*, large mtDNA chromosome; *B*, small mtDNA chromosome). The outer and inner boxes show genes that are transcribed in a clockwise and counterclockwise direction, respectively. Gene color codes are indicated. (*C*) PCR amplifications of the entire small mtDNA using primers indicated by yellow arrows in panel B. Three PCR bands of the expected sizes of 9,911, 9,100, and 10,160 bp were recovered respectively using primer combinations 8.1/6.2, 7.1/8.2, and 6.1/7.2 (table 1). M, molecular marker.

A total of 223,988 reads were produced with an average size of 400 bp. *De novo* DNA sequence assembly of *Rhizophagus* sp. produced 8 contigs ranging from 4,014 to 33,711 bp, containing all the expected mtDNA genes previously recorded in isolates closely related to *R. irregularis*. The sequence coverage of mtDNA contigs ranged between 10 × and 40 × . The mtDNA sequence of *R. irregularis* DAOM 234179 for which SIRs and plasmid-related DNA polymerase (*dpo*) sequences were annotated, was used as a reference for gene synteny and for designing primer sets at the termini of each contig to complete contig assemblies by Sanger sequencing. The eight contigs were assembled in two contigs of 29 and 56 kb. Many attempts were made to assemble the 29-kb contig flanked by *atp6* and *rnl* and the 56-kb contig flanked by *cox2* and *cox1* using the primers listed in table 1. Using conventional and long PCRs, no amplification was observed for any of the four possible primer combinations while positive controls produced fragments of the expected size (data not shown). We therefore attempted to join the termini of each contig with the primers designed at their termini (fig. 1*A*). The combination of the forward *rnl* primer with the reverse *atp6* primer produced an amplicon of 3,309 bp, while a PCR product of 5,690 bp was obtained using the forward *cox1* primer with the reverse *cox2* primer (fig. 1 and table 1). Sanger sequencing of these PCR products confirmed the circularity of the ∼29 and ∼56 kb contigs. To double-check mtDNA circularity, the small mt chromosome was fully amplified using overlapping long-range PCR. Three positions of the small mt chromosome were selected to design three forward primers and their complementary sequences were used as reverse primers (table 1 and fig. 2*B*). The three primer combinations successfully amplified three fragments of the expected sizes covering the entire small chromosome (fig. 2*C*). Taken together, these results confirmed that the mt genome of DAOM 213198 is made of two circular chromosomes (fig. 2), an unusual structure because previously sequenced mt genomes within AMF exhibited a single circular chromosome.

Prior to completing the whole mt maps, the presence of ∼6 kb fragments between *atp8* and *atp9* was validated by PCR and Sanger sequencing approaches, finalizing the larger chromosome size at 61,942 bp. Compared with closely related species, this region contained unexpected *dpo*-like fragments and previously unidentified sequences based on querying GenBank database. These fragments are the major cause of mt genome expansion in the size of *Rhizophagus* sp. DAOM 213198 (figs. 1 and 2). Together, the two mtDNA chromosomes represented 91,042 bp, which is the largest mt genome sequenced so far in *Glomerales* order, while *Gigaspora rosea* has the largest mt genome recorded in *Glomeromycota* (97.3 kb; Nadimi et al. 2012). Together, the two mtDNAs of *Rhizophagus* sp*.* DAOM 213198 contained the 41 mt genes recorded in *R. irregularis* DAOM 234179, consisting of two ribosomal ribonucleic acids (rRNAs), 14 protein-coding genes, and 25 transcribed ribosomal ribonucleic acid (tRNA)-coding genes. The 29-kb mtDNA chromosome contained *rnl*, *atp6*, *nad2*, *nad3*, *nad6*, and *cox3* in addition to five tRNAs and eight plasmid-related DNA polymerases (*dpo*). The 62-kb chromosome contained *rns, atp9*, *nad5*, *cob*, *nad4*, *nad1*, *nad4L*, *cox1*, *cox2*, and *atp8* and harbored 20 tRNAs and 3 *dpo* sequences. Both mt chromosomes showed a low Guanine-Cytosine content of 36.5%.

### Mitochondrial Genome Conversion

**Fig. 3.—:**
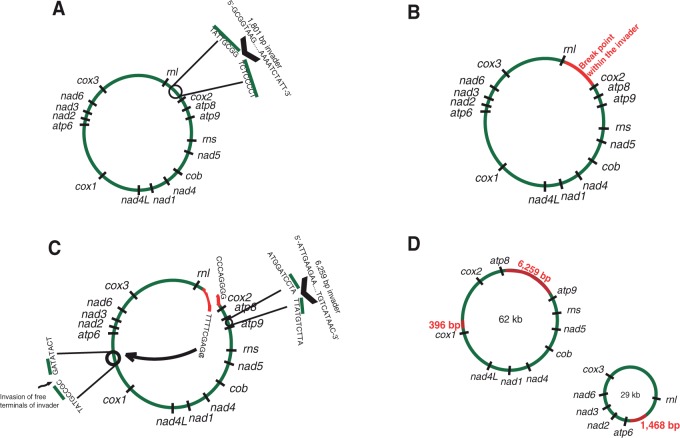
Hypothetical pathway of mtDNA conversion in *Rhizophagus* sp. DAOM 213198. (A) Insertion of 1,801 bp *dpo*-like sequence in the intergene of *rnl-cox2* (or possibly in *atp6-cox1*) in ancestral mtDNA. The circle projection shows the break points with their corresponding terminal sequences, while the black arrow head shows the insertion point. (*B*) Putative ancestral mtDNA shows insertion of an invader sequence (red). (*C*) Breakage of inserted fragment (red) and ligation of its 3′ end to N-terminal sequence of an inserted sequence intergene *cox1-atp6* followed by subsequent ligation of 3′ end of *cox1-atp6* inserted sequence with N-terminal of the *rnl-cox2* broken sequence. Another insertion of a 6,259-bp sequence occurred in *atp8-atp9* intergene. (D) Division of the ancestral mtDNA into two circular-mapping mtDNA in *Rhizophagus* sp. DAOM 213198. Red fragments represent the inserted sequences of 396 and 6,259 bp for the 62-kb fragment and 1,468 bp for the 29-kb fragment.

**Fig. 4.—:**
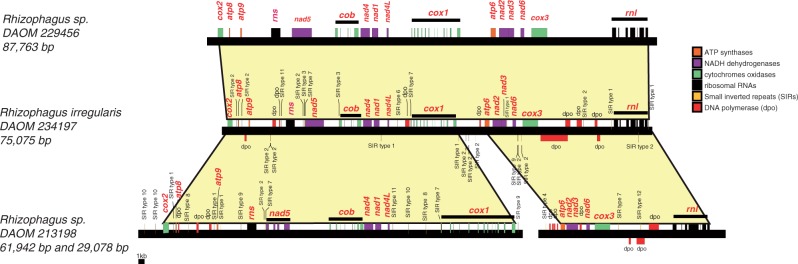
Linear representation of mtDNAs of *Rhizophagus irregularis* DAOM 234179, DAOM 229456, and *Rhizophagus* sp. DAOM 213198 showing their genome synteny. Yellow projections represent mtDNA regions with the same gene order.

Although the gene synteny in *Rhizophagus* sp. DAOM 213198 is similar to *R. irregularis* DAOM 234179 in nonrecombined regions, putative division of the ancestral circular mtDNA created two novel intergenic regions between *rnl*-*atp6* and *cox1*-*cox2* (figs. 3 and 4). The comparative analysis of AMF mt genomes suggests the presence of hot-spot regions subjected to recombination mediated by the integration of *dpo* sequences, such as intergenic regions of *cox3*-*rnl*, *cox1*-*nad4L*, and *rns*-*atp9* (Beaudet, Terrat, et al. 2013).

The comparative sequence analyses of reshuffled intergenic regions of *atp9*-*atp8, rnl*-*atp6*, and *cox1*-*cox2* between the mitochondrial deoxyribonucleic acids of DAOM 213198 and the close relative *R. irregularis* DAOM 234179 suggest that conversion and recombination could act as mechanisms by which the fragmentation of mtDNA occurred in DAOM 213198 (fig. 3). Interestingly, an intergenic region between *cox1* and *cox2* has never been found in any other *Glomeromycota* sequenced so far. The nucleotide sequences of both termini of this unique intergene matched with the putative ancestral intergenes of *rnl*-*cox2* and *cox1*-*atp6* in *R. irregularis* DAOM 234179. This supports the idea that recombination could be initiated by a DSB followed by translocation and reshuffling, resulting in fragmentation of the ancestral mtDNA into two mtDNA circular molecules (fig. 3). Comparison between *rnl*-*atp6* and *cox1*-*cox2* intergenes in DAOM 213198 and *rnl*-*cox2* and *cox1*-*atp6* intergenes in *R. irregularis* DAOM 234179 suggests a *dpo*-like sequence insertion into their common ancestral mtDNA. The inserted *dpo*-like sequence contains some conserved *dpo* domains (which are also found in other mt genomes within Glomeromycetes) flanked by nonidentified sequences according to a TBLASTX search (fig. 3 and supplementary fig. S2, Supplementary Mateiral online). Conserved domains of *dpo*-like sequences at the amino acid level are shown in supplementary figure S3, Supplementary Mateiral online. Figure 3 shows the hypothetical mechanism of the mtDNA fragmentation in DAOM 213198: An insertion of a *dpo* sequence could have occurred in the ancestral intergenic region of *rnl*-*cox2* or *cox1*-*atp6*, followed by a breakdown and disintegration of the inserted *dpo* sequence. Translocation and ligation of breaking sequence termini of *rnl* to *atp6* and *cox1* to *cox2* resulted in the formation of 29- and 62-kb mtDNA circular chromosomes, respectively (fig. 3). The presence of *dpo* fragments in this region strongly suggests a *dpo*-mediated recombination as reported previously (Beaudet, Terrat, et al. 2013). However, the origin of *dpo* domains flanking unknown sequences and their replication and movement mechanisms remain unclear. Comparative genomics analysis between the several AMF mtDNAs in Beaudet, Terrat, et al. (2013) and DAOM 213198 suggests that some intergene regions can be considered as putative hot-spot regions for recombination. Supplementary figure S2, Supplementary Material online summarizes putative recombination hot spots by *dpo*-like sequences (containing the conserved domains) that could impact mt genes reshuffling in *Rhizophagus* sp*.* DAOM 213198 and *R. irregularis* DAOM 234179. This comparative mtDNA analysis provided some clues about the potential mechanisms of genome conversion occurring in the isolate of *Rhizophagus* sp. DAOM 213198.

### SIRs Mediate Recombination in mtDNA

**Fig. 5.—:**
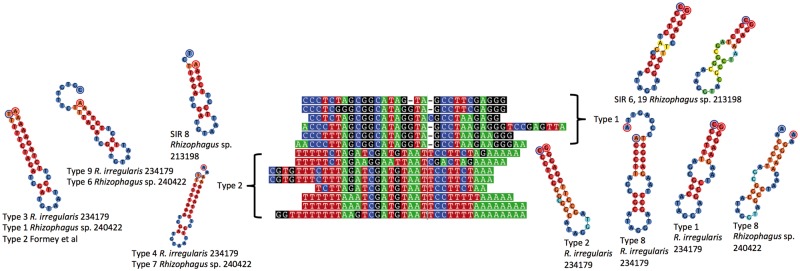
Multiple sequence alignment of the most common identified SIRs. Multiple sequence alignment of 13 types of SIRs found in *R. irregularis* DAOM 234179, *Rhizophagus* sp. DAOM 240422, DAOM 213198, and those reported by Formey et al. (2012). SIR types 1 and 2 are the most frequent SIRs found in all AMF mtDNA analyzed in this study. Secondary structures were predicted based on the energy model of Mathews et al. (2004) and Andronescu et al. (2007). The minimum free energy (MFE) structure of hairpins is colored according to the base-pairing probabilities (red, high; green, mid; blue, low). Blue and red circles around nucleotides represent the beginning and the end of molecules, respectively.

Recombination in mtDNA can also be mediated by short inverted or palindromic repeats, as previously demonstrated (Rattray et al. 2001, 2005). SIRs recorded in DAOM 213198 and in all mtDNAs of *Rhizophagus* spp. publically available (Formey et al. 2012; Beaudet, Terrat, et al. 2013) were analyzed to investigate their role in mtDNAs and their dispersal within and among mt genomes in *Rhizophagus*. On the basis of their DNA secondary structure, a total of 13 types of SIRs were recorded in the mtDNAs of DAOM 213198 (fig. 5 and supplementary fig. S4, Supplementary Material online). The distribution patterns of SIR types, such as types 1 and 2 in *R. irregularis* DAOM 234179, suggest potential repeat-mediated recombination (fig. 4 and table 2). However, the decreased number of some SIRs’ types in mtDNAs of DAOM 213198 that were frequently observed in DAOM 234179 (table 2 and fig. 3) supports their deletion through repeat-mediated recombination as previously reported (Marechal and Brisson 2010; Davila et al. 2011). It remains unclear how such SIR replications and dispersals take place in mt genomes. Presumably, SIRs tend to invade intergenic regions and introns that are under low selective pressure.

**Table 2:** Distribution of SIRs found in *Rhizophagus irregularis* DAOM 234179 and *Rhizophagus* sp. DAOM 213198 Depending on Categorized Types and Genome Localization

*R. irregularis* DAOM 234179			*Rhizophagus* sp. DAOM 213198	
SIR Subtypes	Genome Localization		SIR Subtypes	Genome Localization
T1	*cox1-atp6*		T1	*atp8-atp9*(2)^a^
	*cox1-atp6*			*atp9-rns*(2)
	*nad3-nad6*			*cox2-atp8*(2)
	*cox3-rnl*		T2	*rns-nad5*(2)
	*rnl-cox2*		T4	*cox1* intron(2)
	*atp8-atp9*			*rnl-atp6*(1)^a^
	*nad5 intron*		T7	*rns-nad5*(2)
	*nad1-nad4l*			*nad4l-cox1*(2)
T2	*cox1-atp6*			*cox3-rnl*(1)
	*cox1-atp6*		T8	*nad4l-cox1*(2)
	*nad6-cox3*			*atp8-atp9*(2)
	*nad6-cox3*			*cox3* intron(1)
	*cox3-rnl*		T9	*atp9-rns*(2)
	*cox2-atp8*			*cox1* intron(2)
	*atp9-rns*			*cox1-cox2*(2)
	*atp9-rns*		T10	*nad4l-cox1*(2)
	*rns-nad5*			*cox1-cox2*(2)
	*cob intron*			*cox1-cox2*(2)
T3	*rns-nad5*		T11	*nad4l-cox1*(2)
	*nad5-cob*		T12	*cox3-rnl*(1)
T6	*nad4l-cox3*			
T7	*rns-nad5*			
	*nad4l-cox3*			
T8	*nad6-cox3*			
T11	*atp9-rns*			

^a^Chromosome number is indicated in parentheses.

### Relative Quantification of mtDNA Chromosomes

Real-time quantitative assays showed that the two mtDNAs had a similar abundance (supplementary table S1, Supplementary Material online). This result is surprising because smaller mt genomes usually replicate faster than larger mt genomes (Birky 2001; Schoch and Seifert 2012). A putative mechanism controlling mtDNA replication might occur in the isolate DAOM 213198, resulting in an equal abundance of both mt chromosomes. This has been shown previously (Birky 2001; Schoch and Seifert 2012) where it was found that replication of mtDNAs could be affected by not only the rate of initiation and the rate of completion of mtDNA synthesis but also by the mechanisms that regulate the overall mass of each mtDNA.

### Mitochondrial Inheritance

Mt inheritance and dynamics have been intensively studied in many eukaryotes such as mammals, plants, and yeasts (Birky 2001). However, these aspects have not been investigated in *Glomeromycota*, probably because of their obligate biotrophic lifecycle and their slow growth. Yet, such information about the mitochondrial dynamics and inheritance could give us insights into the evolution of mtDNAs of *Rhizophagus* sp. DAOM 213198. Mt division could produce mitochondria containing both mtDNAs or one of each mtDNA as shown in supplementary figure S5, Supplementary Material online. Mitochondria and their mtDNAs could be randomly passed to the progeny during cell division, as reported by Birky (2001). However, mitochondria are able to undergo fusion followed by fission, thereby regulating their mtDNA segregation (supplementary fig. S5, Supplementary Material online). If such a mechanism of inheritance regulation did not exist, this could lead to forming nonfunctional mitochondria (Chan 2006; Chen and Chan 2009).

Mt genes encoding essential enzymes and subunits for oxidative phosphorylation pathways for energy (ATP) synthase are located in two mtDNAs in *Rhizophagus* sp. DAOM 213198. It is likely that mitochondria lacking one of the two mtDNAs could not be functional. The isolate DAOM 213198 could have a mechanism that controls mtDNA dynamics and inheritance. Without this mechanism, mitochondria with incomplete mtDNA sets could lead to nonfunctional cells (Benard and Karbowski 2009).

## Conclusions

We have documented an unusual mtDNA organization in the isolate *Rhizophagus* sp. DAOM 213198, a close relative to the model species *R. irregularis.* This novel mtDNA feature leads to a new level of understanding of mt genome evolution in eukaryotes. The mtDNA comparative analyses between close relatives of *Rhizophagus* spp. shows that *dpo-*like sequences and SIR-mediated recombinations not only enhance mtDNA reshuffling but could also lead to fragmentation, impacting mobile element dynamics in mt genomes. Mt genomes show a high potential for developing molecular tool kits in order to discriminate isolates and closely related taxa, and to monitor gene exchange and recombination among isolates. Rearrangement of genes and intergenic regions has potential implications in studying population genetics, ecology, and functions of Glomeromycetes in ecosystems. However, the mtDNA organization in two circular chromosomes found in DAOM 213198 raises fundamental questions about their replication and inheritance compared with other AMF harboring one single circular chromosome with a full set of genes. Further investigations are needed to advance our understanding of the evolution of mtDNA in eukaryotes, particularly in basal fungal lineages.

## Supplementary Material

Supplementary figures S1–S5 and table S1 are available at *Genome Biology and Evolution* online (http://www.gbe.oxfordjournals.org/).

Supplementary Data
