# Supplementary material for: The Mitochondrial Genome of the Glomeromycete Rhizophagus sp. DAOM 213198 Reveals an Unusual Organization Consisting of Two Circular Chromosomes
Source: Genome Biol Evol. 2014 Dec 19;7(1):96–105. doi: 10.1093/gbe/evu268 (PMC4316621; doi:10.1093/gbe/evu268)
Supplement: Supplementary Data [file supp_7_1_96__index.html]

The mitochondrial genome of the glomeromycete Rhizophagus sp. DAOM 213198 reveals an unusual organization consisting of two circular chromosomes — The Mitochondrial Genome of the Glomeromycete Rhizophagus sp. DAOM 213198 Reveals an Unusual Organization Consisting of Two Circular Chromosomes — Supplementary Data 

# The Mitochondrial Genome of the Glomeromycete *Rhizophagus* sp. DAOM 213198 Reveals an Unusual Organization Consisting of Two Circular Chromosomes

## Supplementary Data

files

**Files in this Data Supplement:**

- Supplementary Data - docx file
