# Supplementary material for: The Mitochondrial Genome of the Glomeromycete Rhizophagus sp. DAOM 213198 Reveals an Unusual Organization Consisting of Two Circular Chromosomes
Source: Genome Biol Evol. 2014 Dec 19;7(1):96–105. doi: 10.1093/gbe/evu268 (PMC4316621; doi:10.1093/gbe/evu268)
Supplement: Supplementary Data [file supp_evu268_Supplementary_Information.docx]

**Supplementary Material**

**Fig. S1.** Morphological description of spores of *Rhizophagus sp.* DAOM 213198 spores under *in vivo (a. b and c)* and *in vitro (e. f. g and h)* culture conditions. The spore wall (SW) is composed of three layers in both *in vivo* and *in vitro* cultures (a and e). The outer layer SWL1 (b and c) was mucilaginous, evanescent, hyaline and approximately 1 µm thick. The second layer (SWL2) was rigid, smooth, hyaline and approximately 1.5 µm thick. The inner layer (SWL3) was laminate, smooth, pale yellow and approximately 1.5 µm thick. SWL1 and SWL2 were closely attached to each other forming a unique shell, easily detachable from the third layer (SWL3). The subtending hyphae (8.5–9.5 µm) was hyaline and straight and pore open. These images are courtesy of Dr Yolande Dalpé (AAFC, Ottawa. ON).

**Fig. S2.** mtDNA comparative analysis between *R. irregularis* DAOM 234179 and *Rhizophagus* sp. DAOM 213198 isolates. The linear-mapping of mtDNAs containing the newly formed and reshuffled intergenic regions of *rnl*-*atp6* and *cox1*-*cox2* and *atp9*-*atp8* in *Rhizophagus* sp. DAOM 213198 in comparison to its relative *R. irregularis* DAOM 234179. Nucleotide identity comparison with tblastx, between the homologous regions is indicated by the projections. Dark green projections represent sequence identity higher than 90% while light green projections represent sequence identity ranging between 50 and 90%.

**Fig. S3**. Alignment of potentially conserved *dpo*-like translated amino acid sequences from *R. irregularis* DAOM 234179 and *Rhizophagus* sp. DAOM 213198. Distance sequence analysis of *dpo*-like fragments of two closely related species of *R. irregularis* DAOM 234179 and *Rhizophagus* sp. DAOM 213198 reveals that there are some conserved domains in *dpo*s positioned in different intergenic regions.

**Fig. S4**. Multiple sequence alignment of less frequent short inverted repeats. The previously identified SIRs subtypes found in *R. irregulare* DAOM 234179, *Rhizophagus* sp. DAOM 240422 and 213198 (Formey *et al.* 2012) were aligned and classified. Types 3 to 13 represent less frequently found SIRs subtypes in DAOM 213198. Secondary structures were predicted based on the energy model of Mathews *et al.* (2004) and Andronescu *et al.* (2007). The minimum free energy (MFE) structure of hairpins is colored by base-pairing probabilities (red: high; green: mid; blue: low). Blue and red circles around nucleotides represent the beginning and the end of molecule, respectively.

**Fig. S5.** Hypothetical pathway of mtDNA inheritance and dynamics in *Rhizophagus* sp. DAOM 213198. Mitochondrial division could result in the formation of mitochondria containing either mtDNAs or one of each mtDNAs. Fusion dynamic of mitochondria also provide the opportunity for the ones harboring incomplete mtNA to fuse and complete set of mitochondrial genes.

**Table S1**. Absolute quantitative real-time PCR assays performed on DAOM 213198 and DAOM 197198 using *cox1* and *rnl* for the large and small mtDNAs, respectively. PCR efficiencies were 98.33% and 99.86% for *rnl* and *cox1*, respectively.

|  |  | *rnl* quantity | *cox1* quantity | ratio *cox1/rnl* |
| --- | --- | --- | --- | --- |
| 293198 | Dilution 1 | 27 113 | 27 852 | 1.0 |
|  | Dilution 2 | 2 468 | 2 668 | 1.1 |
|  | Dilution 3 | 236 | 291 | 1.2 |
|  | Dilution 4 | 30 | 23 | 0.8 |
| 197198 | Dilution 1 | 3 224 | 2 459 | 0.8 |
|  | Dilution 2 | 295 | 231 | 0.8 |
|  | Dilution 3 | 26 | 20 | 0.8 |
